# Protocol for a systematic review to identify the barriers and facilitators to deliver bystander cardiopulmonary resuscitation (CPR) in disadvantaged communities

**DOI:** 10.1186/s13643-018-0807-5

**Published:** 2018-09-17

**Authors:** Fiona Dobbie, Kathryn Angus, Isabelle Uny, Edward Duncan, Lisa MacInnes, Liz Hasseld, Gareth Clegg

**Affiliations:** 1 Institute for Social Marketing, Faculty of Health Sciences and Sport, University of Stirling, Stirling, FK9 4LA UK; 2 Nursing Midwifery and Allied Health Professionals Research Unit, Faculty of Health Sciences and Sport, University of Stirling, Stirling, FK9 4LA UK; 3 Resuscitation Research Group, The University of Edinburgh, Edinburgh, EH16 4SA UK

**Keywords:** Bystander CPR, Deprived community, CPR training, Social marketing, Social networks, Intervention

## Abstract

**Background:**

A key determinant of survival after out-of-hospital cardiac arrest (OHCA) is bystander cardio pulmonary resuscitation (CPR) which can more than double an individual’s chances of surviving to discharge from hospital. The experience of other international OHCA survival programmes has shown that increasing bystander CPR is strongly associated with an increase in overall survival. However, existing data suggest that the more economically deprived an area is the higher the incidence of cardiac arrest. At the same time, rates of bystander CPR in the same areas are lower, which could result in lower survival rates.

High-profile awareness raising campaigns that are generic focus have not specifically targeted people living in deprived communities who may require more tailored campaigns and interventions to change attitudes and improve confidence to administer bystander CPR. Therefore, this systematic review will explore the facilitators and barriers to engaging with bystander CPR which exist in deprived communities The secondary objective is to identify existing bystander OHCA social marketing and social network intervention campaigns that could inform future activities to improve the rate of bystander CPR in deprived communities.

**Methods:**

Systematic review searching the following databases: CINAHL, MEDLINE, PsycINFO, and Web of Science Core Collection Citation Indexes. Unpublished ‘grey’ literature will also be sourced through web searches, stakeholder interviews, and an advisory group. The reference lists of any relevant reviews will also be checked for additional studies. References will be restricted to those published in 2000 onwards. Authors will independently screen, assess data quality, and extract data for synthesis. A narrative synthesis of study findings will be conducted, with findings presented thematically.

**Discussion:**

This review will focus on all studies that seek to examine the barriers and facilitators to the delivery of bystander CPR in deprived communities and identify examples of previous interventions or activities that could inform the design of a future theory-based intervention to improve the rate of bystander CPR in deprived communities.

**Systematic review registration:**

PROSPERO CRD42017081944

**Electronic supplementary material:**

The online version of this article (10.1186/s13643-018-0807-5) contains supplementary material, which is available to authorized users.

## Background

Out-of-hospital cardiac arrest (OHCA) is a significant health problem in Scotland with approximately 3000 resuscitation attempts each year. Of the 65 people living on Scotland who have attempted resuscitation after OHCA each week, only around 1 in 20 will survive to leave hospital.^1^ This is lower than the 9% survival figure quoted for the rest of the UK and the European average of 1 in 10 [1]. A key determinant of survival after OHCA is bystander cardiopulmonary resuscitation (CPR) which is demonstrated to improve the likelihood of survival to hospital discharge by sevenfold [2]. CPR is an emergency procedure for life support, consisting of manual external cardiac massage to maintain some blood flow to vital organs and can also involve ‘mouth-to-mouth’ ventilation. The experience of other international OHCA survival programmes has shown that increasing bystander CPR improves overall survival [1]. For example, in Sweden three million people, out of a total population of 9.5 million, have been trained in CPR during the last three decades [2]. By 2011, the CPR rate had risen from 31% to amongst the highest in the world at over 70%, with a parallel increase in OHCA survival to 1 month from 5% in 1992 to 11% in 2011 [3]. Similarly, in Denmark, an increase in the bystander CPR rate from 19.4% in 2001 to 43.3% in 2010 was associated with an increase in overall survival to discharge from hospital from 6.5 to 19.1% in the same period [4].

Barriers to administering bystander CPR include a lack of confidence and concerns around causing further harm. Moreover, there is a perception that this fear will be exacerbated in an emergency situation through panic [5]. Existing data suggest that the more economically deprived an area is the higher the rate of cardiac arrest, and crucially, rates of bystander CPR are lower, resulting in lower survival rates [6, 7] . In Scotland, analysis of a linked dataset^1^ of 11,275 OHCA episodes found that between 2011 and 2105, 26.8% occurred in the most deprived areas. The comparable figure for those in the least deprived was 13.9%. Logistic regression (adjusting for age and sex) found that patients in the least deprived areas had a 34% lower risk of death within 30 days after OHCA compared with patients in most deprived. Thus, while large-scale advertising campaigns like the British Heart Foundation’s (BHF) ‘Hands-only CPR’ have a wide reach, more tailored and targeted work is required to improve the rate of bystander CPR in deprived communities [8].

In 2015, the Scottish Government launched Scotland’s Out-of-Hospital Cardiac Arrest strategy. Its ambition is that by 2020 Scotland becomes an international leader in OHCA outcomes [9]. The overall aim of the strategy is to save an additional 1000 lives by 2020, crucial to achieving this is to increase rates of bystander CPR from a baseline of 40% in 2015 [10].

In 2015, a cross-sectional survey of the Scottish population was conducted to explore attitudes, awareness, and perception of bystander CPR. The study concluded that if Scotland is to become an international leader in OHCA outcomes, there is an urgent need for more tailored and targeted interventions to increase rates of bystander CPR. This need is greatest in areas of multiple deprivation. Survey findings also suggest that priority groups include people who are not working, those in a lower social grade, and the elderly. (Dobbie et al. 2018 in press).

## Review aim and research objectives

The aim of the review is to explore the facilitators and barriers to engaging with bystander CPR which exist in deprived communities. Results from this systematic review will inform a research study that seeks to develop a tailored and targeted strategy to improve the rate of bystander CPR in deprived communities in the UK.

The review will address the following research objectives:

1. Identify the barriers perceived by individuals in socioeconomically deprived circumstances to engage with bystander CPR
2. Identify the facilitators perceived by individuals in socioeconomically deprived circumstances to engage with bystander CPR
3. To identify examples of projects, initiatives and activities that have attempted to improve rates of bystander intervention in deprived communities during an out-of-hospital cardiac arrest using social marketing or social network intervention approaches

We are defining the term ‘engaging with bystander CPR’ as a willingness/confidence to learn CPR, perform bystander CPR, and teach/encourage others to learn/engage with bystander CPR.

## Methods

This protocol has been designed using the PRISMA-P guidelines for systematic review protocol development [11].

### Search strategy

Studies for review will be identified from searches of academic literature databases (CINAHL, MEDLINE, PsycINFO, Web of Science Core Collection Citation Indexes). Unpublished ‘grey’ literature will also be sourced through web searches, stakeholder interviews (from the wider project), and an advisory group. The reference lists of any relevant reviews will also be checked for additional studies.

The search strategy will be made up of three concepts. Search terms for (1) out-of-hospital cardiac arrest and cardiopulmonary resuscitation will be combined with search terms for (2) bystanders (including witness, layperson) and search terms for (3) socioeconomically deprived circumstances, see Additional file 1 for a draft search strategy.

References will be restricted to those published in 2000 onwards

### Inclusion/exclusion criteria

The search will include all study types that collected primary data (e.g. qualitative and mixed methods studies as well as quantitative designs) or ran a new analysis of an existing primary dataset, conference abstracts, and unpublished ‘grey’ literature. It will include conference abstracts and unpublished ‘grey’ literature. Studies published in any language will be included, although search terms will be in English. It will exclude systematic reviews, evidence-based guidelines, and literature reviews, but references will be checked. Commentaries and opinion pieces will also be excluded.

### Participants/population

The search will include the following:

- Any study that reports findings for anyone experiencing socioeconomically deprived circumstances, identified used a recognised indicator of deprivation (e.g. Indices of Multiple Deprivation). Studies will be included if the whole sample is a socioeconomically deprived population or area or if the study segments findings by a socioeconomic indicator.
- Any definition of socioeconomic deprivation (including, but not limited to, educational status, employment status, income, occupation, poverty, social change, social class, social condition, neighbourhood/area status)
- In order to review evidence from countries of similar high-income economic backgrounds to the UK and that may also have a socioeconomic inequalities gap, studies must be conducted in any member country of the OECD (Organisation for Economic Co-operation and Development) http://www.oecd.org/about/membersandpartners
- Any age group

The search will exclude those trained and certified in CPR as part of their professional, statutory, or voluntary roles.

### Intervention(s) and exposure(s)

To meet research question 3, the review will include any type of campaign, project or activity that uses a social marketing [12] or social network [13] approach in an attempt to improve the rate of bystander intervention during an out-of-hospital cardiac arrest in deprived communities. Bystander intervention can include telephoning for help and performing CPR (with or without a public access defibrillator/emergency access defibrillator). Studies that only address bystander use of defibrillation (PADs, EADs) will not be included.

### Comparator(s)/control

Any project, initiative, or activity that aims to improve rates of bystander CPR, or no intervention, is treated as a control.

### Outcomes

The primary outcome will be, from a potential OHCA bystander’s perspective, to explore any barriers and facilitators in performing bystander CPR, to learn bystander CPR, or to enable others to learn.

Secondary outcomes will be any social marketing and social network intervention elements and approaches used, as well as any measures of the effectiveness of social marketing and social network interventions.

## Data management

Once the search terms have been piloted and finalised, electronic databases will be searched and the references will exported to EndNote bibliographic software for storage and for removal of duplicates. After removing duplicates, titles and abstracts will be reviewed to identify relevant studies using a pre-defined screening checklist based on the inclusion/exclusion criteria described in the previous section. A batch of 10% of the review’s search results will be screened in triplicate (KA, IU, and FD) to test shared understanding and application of the screening checklist. If agreement is low (< 80%), a further batch of results will be triple-screened and an explanatory text will be added to the checklist questions to assist with consistent screening. If necessary, the process will be repeated until there is agreement on at least 80% records between reviewers, then the three reviewers will single-screen a portion of the remaining title and abstract records each.

Full papers will be retrieved for studies deemed potentially relevant. Double-screening of full papers will be conducted (KA and IU), and those deemed irrelevant will be removed. Where two reviewers disagree, a third reviewer (FD) will screen the full paper for inclusion or exclusion. Thereafter, a final list of studies for full review will be generated.

## Quality assessment

A standard appraisal checklist appropriate to the study design (e.g. Critical Appraisal Skills Programme http://www.casp-uk.net/checklists) will be used independently by two members of the review team (adjudicated by a third). Each study will be graded according to the validity of the study and usefulness of its results to the review.

## Strategy for data collection and synthesis

Data will be extracted from all studies (regardless of the evidence grading) and evidence tables produced by one team member, checked for accuracy by a second. Evidence tables will summarise each study’s: aim, sample, design, county of origin, key barriers/facilitators findings, and quality grading. Tables will summarise any social marketing and social network intervention elements and approaches used.

A broader 2015 research review of the literature (Stirzaker R, Smith C: Improving Bystander CPR in Out of Hospital Cardiac Arrest (OHCA) in Scotland: A Research Review, unpublished) demonstrated that given the range outcome measures, a meta-analysis of extracted data will be unlikely to be feasible. Instead, the anticipated heterogeneous, largely qualitative and observational data will be synthesised in a narrative format focused around the review’s objectives, with findings presented thematically.

## Discussion

We could not identify a published systematic review examining the barriers and facilitators to bystander CPR in deprived communities. This review will focus on all studies conducted in OECD countries published since 2000 that seek to examine the barriers and facilitators to the delivery of bystander CPR in deprived communities and identify examples of previous intervention/activities that may inform the design of future theory-based interventions in improving the rate of bystander CPR in deprived communities.

There are limitations to the outlined systematic review. The review is part of a larger study to develop an intervention to improve the rate of bystander CPR in the UK. This means we have limits on the scale of the review due to resources and to be relevant to the aim of the larger study. Our limitations on publication date and included countries may result in some relevant, but ineligible, studies being missed from the synthesis, potentially introducing bias to our review. We also anticipate having to collect a high number of articles for full-text review due to the limited detail on socioeconomic status and deprivation in many papers’ titles, abstracts, and keywords.

## Additional file

Additional file 1: Draft search strategy for MEDLINE (OVID). (DOCX 19 kb)

## Abbreviations

CINAHL: Cumulative Index to Nursing and Allied Health Literature
CPR: Cardiopulmonary resuscitation
OECD: Organisation for Economic Co-operation and Development
OHCA: Out-of-hospital cardiac arrest
PRISMA-P: Preferred Reporting Items for Systematic review and Meta-analysis Protocols

## Footnotes

1 Unpublished data from the Resuscitation Research Group (RRG) at the University of Edinburgh.
