# Supplementary material for: Protocol for a systematic review to identify the barriers and facilitators to deliver bystander cardiopulmonary resuscitation (CPR) in disadvantaged communities
Source: Syst Rev. 2018 Sep 17;7:143. doi: 10.1186/s13643-018-0807-5 (PMC6142639; doi:10.1186/s13643-018-0807-5)
Supplement: Supplementary file 1 — Draft search strategy for MEDLINE (OVID). (DOCX 19 kb) [file 13643_2018_807_MOESM1_ESM.docx]

Additional file 1: MEDLINE example full search strategy. This search strategy will be adapted for each database.

| 1. exp Cardiopulmonary Resuscitation/ |
| --- |
| 2. exp Out-of-Hospital Cardiac Arrest/ |
| 3. Defibrillators/ |
| 4. Electric Countershock/ |
| 5. heart arrest$1.mp. |
| 6. cardiac arrest$1.mp. |
| 7. cardiopulmonary resuscitation.mp. |
| 8. cardio-pulmonary resuscitation.mp. |
| 9. cardio pulmonary resuscitation.mp. |
| 10. CPR.mp. |
| 11. mouth-to-mouth.mp. |
| 12. mouth to mouth.mp. |
| 13. cardiac massage.mp. |
| 14. heart massage.mp. |
| 15. chest compression$1.mp. |
| 16. out of hospital.mp. |
| 17. out-of-hospital.mp. |
| 18. OHCA.mp. |
| 19. (automat* adj2 (external defibrillator$1 or external defibrillation)).mp. |
| 20. (defibrillat* adj2 public access).mp. |
| 21. 1 or 2 or 3 or 4 or 5 or 6 or 7 or 8 or 9 or 10 or 11 or 12 or 13 or 14 or 15 or 16 or 17 or 18 or 19 or 20 |
| 22. Community Networks/ |
| 23. exp Voluntary Health Agencies/ |
| 24. Volunteers/ |
| 25. witness*.mp. |
| 26. bystander$1.mp. |
| 27. volunteer*.mp. |
| 28. voluntary.mp. |
| 29. layperson$1.mp. |
| 30. laypeople.mp. |
| 31. lay person$1.mp. |
| 32. lay people.mp. |
| 33. lay-person$1.mp. |
| 34. lay-people.mp. |
| 35. (lay adj1 rescue$2).mp. |
| 36. lay-rescuer$1.mp. |
| 37. nonprofessional$1.mp. |
| 38. non-professional$1.mp. |
| 39. unskilled.mp. |
| 40. untrained.mp. |
| 41. First Aid/ |
| 42. first aid.mp. |
| 43. 22 or 23 or 24 or 25 or 26 or 27 or 28 or 29 or 30 or 31 or 32 or 33 or 34 or 35 or 36 or 37 or 38 or 39 or 40 or 41 or 42 |
| 44. exp Health Services Accessibility/ |
| 45. Health Status Disparities/ |
| 46. exp Residence Characteristics/ |
| 47. Digital Divide/ |
| 48. exp Socioeconomic Factors/ |
| 49. class.mp. |
| 50. demographic*.mp. |
| 51. deprivation.mp. |
| 52. deprive*.mp. |
| 53. disadvantage*.mp. |
| 54. disparit*.mp. |
| 55. economic.mp. |
| 56. education*.mp. |
| 57. employed.mp. |
| 58. employment.mp. |
| 59. income.mp. |
| 60. inequalit*.mp. |
| 61. occupation*.mp. |
| 62. post-code*.mp. |
| 63. postcode*.mp. |
| 64. (postal adj code*).mp. |
| 65. poverty.mp. |
| 66. SES.mp. |
| 67. (social adj1 (class or factor or factors)).mp. |
| 68. socioeconomic.mp. |
| 69. socio-economic.mp. |
| 70. unemploy*.mp. |
| 71. zipcode*.mp. |
| 72. (zip adj code*).mp. |
| 73. 44 or 45 or 46 or 47 or 48 or 49 or 50 or 51 or 52 or 53 or 54 or 55 or 56 or 57 or 58 or 59 or 60 or 61 or 62 or 63 or 64 or 65 or 66 or 67 or 68 or 69 or 70 or 71 or 72 |
| 74. 21 and 43 and 73 |
| 75. ..l/ 74 yr=2000-2018 |
